# Supplementary material for: Patient perceptions of the impact of inducible laryngeal obstruction on quality of life
Source: PLoS One. 2024 Jul 16;19(7):e0307002. doi: 10.1371/journal.pone.0307002 (PMC11251631; doi:10.1371/journal.pone.0307002)
Supplement: Attachment — Submitted filename: Response to Reviewers.docx [file pone.0307002.s001.docx]

Dear Reviewers,

We are grateful for the time and expertise you have brought to reviewing this work. We have provided a response to each required and recommended item. We hope that the enclosed responses, and where applicable, the revisions within the manuscript, are acceptable.

**Regarding revisions required for acceptance:**
1. Provide more descriptive details on the study population, including Exercise-ILO, asthma, COPD, mental health disorders, medications, etc.

- Thank you for the feedback. Additional information regarding prevalence of asthma, COPD, mental or behavioral health diagnoses, and inhaler use have been added to the manuscript in response to this feedback. The data added are summarized here:

| **Airway and Respiratory History** | | | |
| --- | --- | --- | --- |
| Asthma | COPD | OSA | Current inhaler use |
| 14 (53.8%) | 2 (7.7%) | 3 (11.5%) | 13 (50%) |
| **Mental/Behavioral Health Diagnoses** | | | |
| Any | Anxiety | Depression |  |
| 11 (42.3%) | 7 (26.9%) | 8 (30.8%) |  |

- Regarding the request to differentiate E-ILO from other cases of ILO, the results section (lines 157-159) does identify the proportion of participants who described exercise as a trigger, and the proportion of participants who ONLY described exercise as a trigger. One could interpolate the E-ILO diagnosis from that however, at the time of the interviews, E-ILO was not commonly described in clinical settings as a distinct entity and so we did not have specific E-ILO diagnoses tracked as a data point.

2. Re-phrase lines 90-92 to clarify the term 'exclusion diagnosis'.

- Thank you for your feedback. Lines 90-92 have been edited to better clarify the way the term exclusion diagnosis has been used within the context of ILO and to further indicate why cases with certain diagnoses would be appropriate to include in this context. (This now appears as 103-110 due to the volume of edits).

3. Describe the medical workup for patients, including consultations with ENT specialists and pulmonologists.

Thank you, the following text has been added to the methods section:

*Patients were referred from a variety of different specialties including primary care, pulmonology, asthma/allergy. Assessment of laryngeal appearance and function was completed using videostroboscopy as standard of care at all evaluations. Given that ILO can be described disorder of laryngeal behavior or function and because patients have already seen many specialities to rule out other diagnoses, adult patients were routinely evaluated and treated in our clinic by SLP. Occasionally, they were scheduled in multi-disciplinary visits with ENT.*

4. Clarify the sentence in lines 38-39 of the abstract.

- 4 Thank you for the feedback. Additional wording was included to resolve ambiguity about the patient demographic data.

5. State the themes and subthemes directly in the abstract.

- Thank you for the feedback. In this case, themes have been listed in the abstract. The wording introducing them has been adjusted and italics have been added in an attempt to make this more clear. As there were 54 subthemes identified, I believe it would not be possible or practical to list all of these in the abstract. We have updated the abstract to indicate that 54 were identified in the hopes this is sufficient. They do appear in the appended table.

6. Revise line 74 in the introduction to better reflect the subject matter.

- Line 74 has been revised to better reflect the subject matter.

7. Start the methods section with a statement of study design and setting.

- Thank you for the feedback. The methods section has been edited accordingly.

8. Define eligible participants before discussing selection methods.

- Updated accordingly, thank you.

9. Specify where potential eligible participants were found.

- Thank you, participant selection section has been revised to include this.

10. Define exclusion criteria for the study.

- Thank you, this has been revised accordingly.

11. Clarify the transcription process in the methods section.

- This has been clarified, thank you.

12. Replace "primary investigator" with initials in the methods section.

- Thank you, this has been completed.

13. Clarify the term "7 additional themes" in the results section.

- We have attempted to address this. Your feedback points out the ambiguity here. Thank you.

14. Replace the term "subjects" with "participants" or "respondents" in the results section.

- Thank you for pointing out the use of subject. It has been replaced with participant in all cases.

15. Include quotes from respondents to validate themes in the results section.

- Thank you for the feedback. Quotes have been added to the sections on physical and emotional impacts. Respondent quotes had not previously been included in those sections the way they were in others. We hope this helps to better validate these themes.

**Regarding other recommended revisions:**
1. Describe how patients presented, such as MTD/voice, breathlessness, ‘asthma’, chronic cough, irritant-induced, etc.

Thank you for your feedback. We clarified that dyspnea was the chief concern for all participants but that others (as the analysis shows) did have comorbid voice and cough complaints.

2. Consider using VCD/ILO or at least mentioning it for clarity.

We respectfully decline to put VCD in the manuscript. We feel the evolution away from use of the term VCD is well documented in related literature and we note that databases now cross reference ILO with VCD and PVFM.

3. Indicate if any questionnaires were administered to shed light on the (poly)symptomatic burden.

Thank you for the feedback. The open-ended nature of the interviews allowed for patients to speak to polysymptomatic burden.

Participant reports to this effect are included in the table. An example of this might include the following quote under the theme *health and prognosis*:

They tell me I’ve got arthritis; I’ve got fibromyalgia, they tell me this and that I’m just like “really?” I don’t want it, take it away.

In this case, the subtheme was entitled “other factors or comorbidities.” Polysymptomatic burden would have been a great name for this subtheme, but that specific title did not arise from the coding process.

4. Provide frequency or proportion of the various subthemes in the thematic analysis.

Thank you for the feedback. This had been completed in some areas of the analysis (for example, the section on symptoms and triggers) In an effort to summarize the data a succinctly as possible, examples of more common sub-themes are included in Table 2.

5. Create a figure to summarize the work.

This is difficult for us to interpret without more detail and so no additional figures were added at this time.

6. Clarify the phrase "Participation perception of health and overall prognosis" in the abstract.

Thank you for the feedback. Our apologies, the intended text was “participant perception of health and overall prognosis.” The error has been fixed in the manuscript. The theme is intended to code content regarding how participants perceive their health and prognosis.

7. Revisit the conclusion in the abstract to align with the results.

In response to this feedback, we have made some minor revisions to the conclusion in the abstract. It now states “patients appear to place particular emphasis on the emotional and psychosocial consequences of ILO as well as factors that ameliorate the condition…

We hope that these changes better reflect the results in the manuscript. Thank you.

8. Revise the subheading "semi-structured interviews" to "Data Collection."

Completed, thank you.

9. Describe the role of a "speech-language pathologist" in the study.

Thank you for your feedback. In addition to the description about speech-language pathologists conducting the interviews and analysis in the study, the methods section now includes additional details about the clinic model and the role of the SLP in diagnosis and management.

10. Clarify the process of devising categories in the methods section.

Thank you for this feedback. As Burnard’s method was utilized and cited, we hope that the cited publication could be referred back to for further info on the process of devising categories.

11. Move lines 124-126 to the methods section under data analysis.

- Thank you for the feedback. After attempting to make the suggested edits, we felt the lines made the most sense included under the new heading “data collection.”
