# Supplementary material for: Patient perceptions of the impact of inducible laryngeal obstruction on quality of life
Source: PLoS One. 2024 Jul 16;19(7):e0307002. doi: 10.1371/journal.pone.0307002 (PMC11251631; doi:10.1371/journal.pone.0307002)
Supplement: Attachment — Submitted filename: Respose to Reviewers.docx [file pone.0307002.s002.docx]

March 26^th^ 2025

Dear Esteemed Reviewers,

We thank you for your continued time and attention in the review of this work. Your comments and insight have guided us in the revision process such that we hope the result is a manuscript that reflects your feedback and would better communicate our work, findings and their relevance to this journal’s readership.

The remaining reviewer comments and our responses are included below.

With Gratitude,

Katherine McConville & Susan L. Thibeault

**Reviewer #1**

- Comments addressed, thank you. No further comments. Although the authors are right that "ILO" is supplanting "VCD", many North Americans (including recent peer reviewed work), primary care docs, many patients, and most critically, people who are stumbling across the field for the first time, will look up or use "VCD". I still strongly suggest putting "VCD" in the abstract to enhance the visibility of the work and then you can clarify ILO at first mention. Otherwise, a large portion of potential readership may miss your very important findings!

Response: *We truly appreciate your review. Thank you for your thoughtful comments and guidance!*

**Reviewer #2:**

- You probably want to follow the scientific journal format. For example, under methods, the section opens with a statement on ethics approval rather than study setting and design.

Response: *Thank you for your feedback. After looking over several recent articles in this publication, we have changed the placement of the ethics approval statement to mirror its placement in similar published articles. We have also revised our user of headers to better follow the specified format.*

- I also find certain important aspects of methods either missing or clumped where they should not be; the section labelled “semi-structured interviews” has in it (1) participants’ recruitment producers, (2) data collection, (3) data analysis. This makes the review process challenging because matters are where they should not be and they are brief, leaving readers unable to see through what exactly was done and how they were done. I find two clan copies and one copy with track changes. The second clean copy appears to reflect the revised manuscript. But it still not following the manuscript style. For example, data collection subheading has in it data analysis as well. I therefore stopped my review at the methods section. Otherwise, find my specific comments on the abstract below.

Response: *Thank you for pointing this out. We have reorganized some of the content in the “Materials and methods” section and revised our user of headers and the format of these headers to follow the specified format and hopefully improve readability.*

- Abstracts – background
  You may want to take note of the expression, “…in a meaningful proportion…” It is difficult to understand what you exactly mean. Kindly use formal scholar expression to say the same.

*Response: Thank you for the thoughtful feedback. We have changed the description to “noteworthy” and hope this is acceptable. Our rationale for the use of “meaningful” had been that we felt it conveyed that this specific disorder contributes to dyspnea in a noteworthy proportion of patients with dyspnea. “Significant” was avoided as we are not speaking to statistical significance and wish to prevent confusion. “Large” was not selected as it might infer a majority and the literature shows that this does not make up a majority of these cases. For example, 22% of patients with frequent emergency room visits due to sudden onset dyspnea are suspected to have ILO.*

- Abstract – Methods: The statement, “Transcription underwent content analyses across multiple raters until consensus regarding analyses was reached”. It is again quite challenging for me to understand the message you are communicating here. You may want to revise it. So did you use content analysis approach to the data/transcript? Was the consensus on codes or themes? When you talk of raters, it is challenging to get what you mean in the sense of qualitative analyses approaches.

*Response: We value your feedback and expertise. We have included more detail in the abstract that identifies the published method we used. We hope this provides more clarity in the abstract. The methods are described in more detail in the body of the manuscript.*

- Abstract – Results: The statement, “Consistent with literature . . . with ILO…” This is the style for discussion where you make comparisons of your findings with existing literature and yet here you are reporting results of this study. You may want to revise accordingly.

*Response: Thank you for the feedback. The preponderance of female cases of ILO is so notorious in the literature on this disorder that it seemed strange not to acknowledge that this was expected. Your feedback makes clear that it is not appropriate in the abstract, and it has been removed.*

- Lines 38 – 39; “Patients description . . . in all interviews”. What is the message in this statement? Kindly state it differently so that it becomes easy for an ordinary reader like myself to peak up the message. You may for example want to provide exactly what their descriptions of symptoms were i.e. you provide the themes and or subthemes on symptoms.

*Response: We have clarified that “symptoms and triggers” was judged to be a theme in the interview data and why. We appreciate this feedback and hope it improves the readability of this section.*

- Again, lines 40 – 41; “Seven additional themes . . . the interview material”. The immediate question is – additional to which ones? And one does not see fore to this statement any descriptions of themes! Kindly present the themes and subthemes under your results section, and among the themes/subthemes there will be those on symptoms at presentation.

*Response: Thank you for the feedback. We believe the revisions made in lines 38-39 now help clarify this as “symptoms and triggers” was labeled the first theme.*

- Line 42; “. . . participation perception . . .” This expression is not regularly used in ordinary academic discourse. Kindly rephrase so that you clearly bring out what you indeed wish to communicate. In the current form, an ordinary reader like me fails to get the message.

*Response: We have clarified that “health and prognosis” was a theme extracted from the interviews. The expression triggering confusion was intended to convey that the participant’s perspectives regarding health and prognosis were a theme. We hope the new wording better conveys this and we thank you for the feedback.*

- Conclusions: Your emphasis is on the consequences of ILO diagnosis, especially social consequences. That is alright. However, you may want to have issues on consequences directly or indirectly alluded to in the result section, and should appear to carry a high significance so that it becomes the major issue in the conclusion.

*Response: Thank you for the feedback. We hope that some revisions made to both the results and the conclusions sections address this concern.*
