# Supplementary material for: Patient perceptions of the impact of inducible laryngeal obstruction on quality of life
Source: PLoS One. 2024 Jul 16;19(7):e0307002. doi: 10.1371/journal.pone.0307002 (PMC11251631; doi:10.1371/journal.pone.0307002)
Supplement: Attachment — Submitted filename: Response to Reviewers.docx [file pone.0307002.s003.docx]

June 26^th^ 2024

Dear Esteemed Reviewers,

We thank you for your continued time and attention in the review of this work. Your comments and insight have guided us in the revision process such that we hope the result is a manuscript that reflects your feedback and would better communicate our work, findings and their relevance to this journal’s readership.

The remaining reviewer comments and our responses are included below.

With Gratitude,

Katherine McConville & Susan L. Thibeault

Reviewer #1:

 Nil further comments. Thank you. Revision is satisfactory and I think it is suitable for publication.

**Response: Thank you again for your time and attention to this work.**

Reviewer #2: 

This manuscript has been greatly improved. Congratulations. You can improve on it further by revising it alongside the journal format and style especially the materials and methods section.

**Response: Thank you, please see additional responses and revisions in an effort to achieve this.**

The ethical considerations is included under "semi-structured interviews".

**Response: Thank you for pointing this out. Ethical considerations are now included under a unique heading. We believe this new section also adds some context that may address your concerns about recruitment (though this is further addressed in the discussion section now based on your feedback).**

The study design is missing. What is this study design? Is it phenomenology? The first statement under materials and methods is on data collection when it should indeed start with study design and setting.

**Response: Thank you for your feedback. We have clarified study design.**

Under participant recruitment and selection, you state that recruitment was conducted by the treating clinician. Why was recruitment done by clinicians and not the research team? You may want to discuss the power imbalance between patient and treating physicians with respect to accepting participation and consent as well as discuss the influence of this power imbalances on the quality of the data.

**Response: Thank you for raising this excellent point. In response to your feedback, we have clarified that the treating clinician recruited participants but did not conduct the interview out of concern this would bias responses.**

**Our discussion section now further explains that recruitment patterns had to work around our busy clinic model with limited resources for a constant onsite research support team.**

**Your additional points about power imbalance are well-taken and we have tried to address these further in the discussion as well.**

I am quite comfortable with the results and discussions sections.

**Response: We sincerely appreciate your feedback.**
